# The sphingosine 1-phosphate receptor 2/4 antagonist JTE-013 elicits off-target effects on sphingolipid metabolism

**DOI:** 10.1038/s41598-021-04009-w

**Published:** 2022-01-10

**Authors:** Melissa R. Pitman, Alexander C. Lewis, Lorena T. Davies, Paul A. B. Moretti, Dovile Anderson, Darren J. Creek, Jason A. Powell, Stuart M. Pitson

**Affiliations:** 1 Molecular Therapeutics Laboratory, Centre for Cancer Biology, University of South Australia and SA Pathology, Adelaide, Australia; 2 School of Biological Sciences, University of Adelaide, Adelaide, Australia; 3 Drug Delivery, Disposition and Dynamics, Monash Institute of Pharmaceutical Sciences, Monash University, Parkville, VIC Australia; 4 Adelaide Medical School, University of Adelaide, Adelaide, Australia

**Keywords:** Lipids, Cell signalling

## Abstract

Sphingosine 1-phosphate (S1P) is a signaling lipid that has broad roles, working either intracellularly through various protein targets, or extracellularly via a family of five G-protein coupled receptors_._ Agents that selectively and specifically target each of the S1P receptors have been sought as both biological tools and potential therapeutics. JTE-013, a small molecule antagonist of S1P receptors 2 and 4 (S1P_2_ and S1P_4_) has been widely used in defining the roles of these receptors in various biological processes. Indeed, our previous studies showed that JTE-013 had anti-acute myeloid leukaemia (AML) activity, supporting a role for S1P_2_ in the biology and therapeutic targeting of AML. Here we examined this further and describe lipidomic analysis of AML cells that revealed JTE-013 caused alterations in sphingolipid metabolism, increasing cellular ceramides, dihydroceramides, sphingosine and dihydrosphingosine. Further examination of the mechanisms behind these observations showed that JTE-013, at concentrations frequently used in the literature to target S1P_2/4_, inhibits several sphingolipid metabolic enzymes, including dihydroceramide desaturase 1 and both sphingosine kinases. Collectively, these findings demonstrate that JTE-013 can have broad off-target effects on sphingolipid metabolism and highlight that caution must be employed in interpreting the use of this reagent in defining the roles of S1P_2/4_.

## Introduction

The sphingolipid pathway is comprised of a range of bioactive lipids that contribute to cellular membranes and can act as signaling molecules. Sphingolipids are produced predominantly in the endoplasmic reticulum but can be trafficked and modified in various cellular locations such as the plasma membrane, lysosomes, mitochondria and the cytoplasm. The cellular balance of the pro-apoptotic lipids, ceramides and sphingosine, and the pro-survival sphingosine 1-phosphate (S1P), produced by the sphingosine kinases (SKs), can impact on cell fate and has been coined the “sphingolipid rheostat”^1^. S1P can bind and regulate a range of intracellular targets or be exported from cells to act as a ligand for a family of five G-protein coupled receptors; S1P_1–5_^2^. The S1P receptors have been a focus of interest due to their involvement in most organ systems, with vital roles during embryonic development, immune cell trafficking, maintenance of physiological homeostasis and their contribution to a broad range of pathogenic processes^3^. In particular, the discovery of novel agents that target S1P_1_ drove an intense focus on the role of S1P_1_ in lymphocyte trafficking and led to several FDA approved S1P_1_ modulating agents^4^, most notably Fingolimod (FTY720)^5^, for the management of multiple sclerosis. By comparison, agents that selectively target the other receptors are less abundant and less well characterized^6^.

JTE-013 is one of the only known, and most widely used S1P_2_ antagonist^7^. It has a reported low nanomolar affinity for S1P_2_ (IC_50_ 17 ± 6 and 22 ± 9 nM for human and rat S1P_2_, respectively) and showed selectivity against S1P_1_ or S1P_3_ at concentrations up to 10 μM in CHO cells expressing the receptors^8,9^. Subsequently, however, JTE-013 was reported to also act as an antagonist of S1P_4_, although with somewhat lower affinity (IC_50_ of 237 nM)^10^. Despite its reported high potency in targeting S1P_2_ (and S1P_4_), throughout the literature JTE-013 has been frequently employed as a selective targeting agent for S1P_2_ at doses normally in the micromolar range, with concentrations as high as 20 μM^11–15^. The specificity of JTE-013 has been previously brought into question, with Salomone et al. demonstrating that it blocked S1P-induced vasoconstriction in S1P_2_ knockout mice and was also effective at blocking vasoconstriction induced by the prostanoid analog U46619, endothelin-1 or high KCl^15^. Furthermore, Li et al. showed that JTE-013 enhanced excitability in neuronal cells even though they lacked S1P_2_ expression^16^. While these studies called into question the selectivity, and thus the usefulness of JTE-013 as a tool to probe the functions of S1P_2_^15,17^, debate remains if these observations arose due to “off-targets” of JTE-013 (which were not definitively identified), or from the complex pharmacology of G protein-coupled receptors^18^.

Our recent work demonstrated that JTE-013 induced the loss of the important pro-survival protein Mcl-1 in acute myeloid leukaemia (AML) cells, recapitulating the results seen with SK1 inhibition, and suggested an oncogenic role for S1P_2_ downstream of SK1 in this malignancy^13^. In an effort to further probe our observations with JTE-013 in AML, we have now used mass spectrometric lipidomics to assess sphingolipid levels in response to AML cell treatment with JTE-013. Our analysis revealed that JTE-013 caused significant alterations in sphingolipid metabolism, increasing cellular ceramides, dihydroceramides, sphingosine and dihydrosphingosine. Our further analysis demonstrated that these effects were due, at least in part to JTE-013 directly inhibiting dihydroceramide desaturase 1 (Des1), SK1 and SK2. Notably, inhibition of these enzymes by JTE-013 occurs at concentrations previously used in many published studies for selective targeting of S1P_2_ (10 µM). Collectively these findings suggest considerable caution is required in the interpretation of findings gained from the use of JTE-013, particularly at higher concentrations, and that some previous findings may warrant further investigation to distinguish the effects of S1P_2_ antagonism versus other enzymes in the sphingolipid pathway such as Des1 and the SKs.

## Results

### JTE-013 alters sphingolipid metabolism in MV411 AML cells

**Figure 1:**
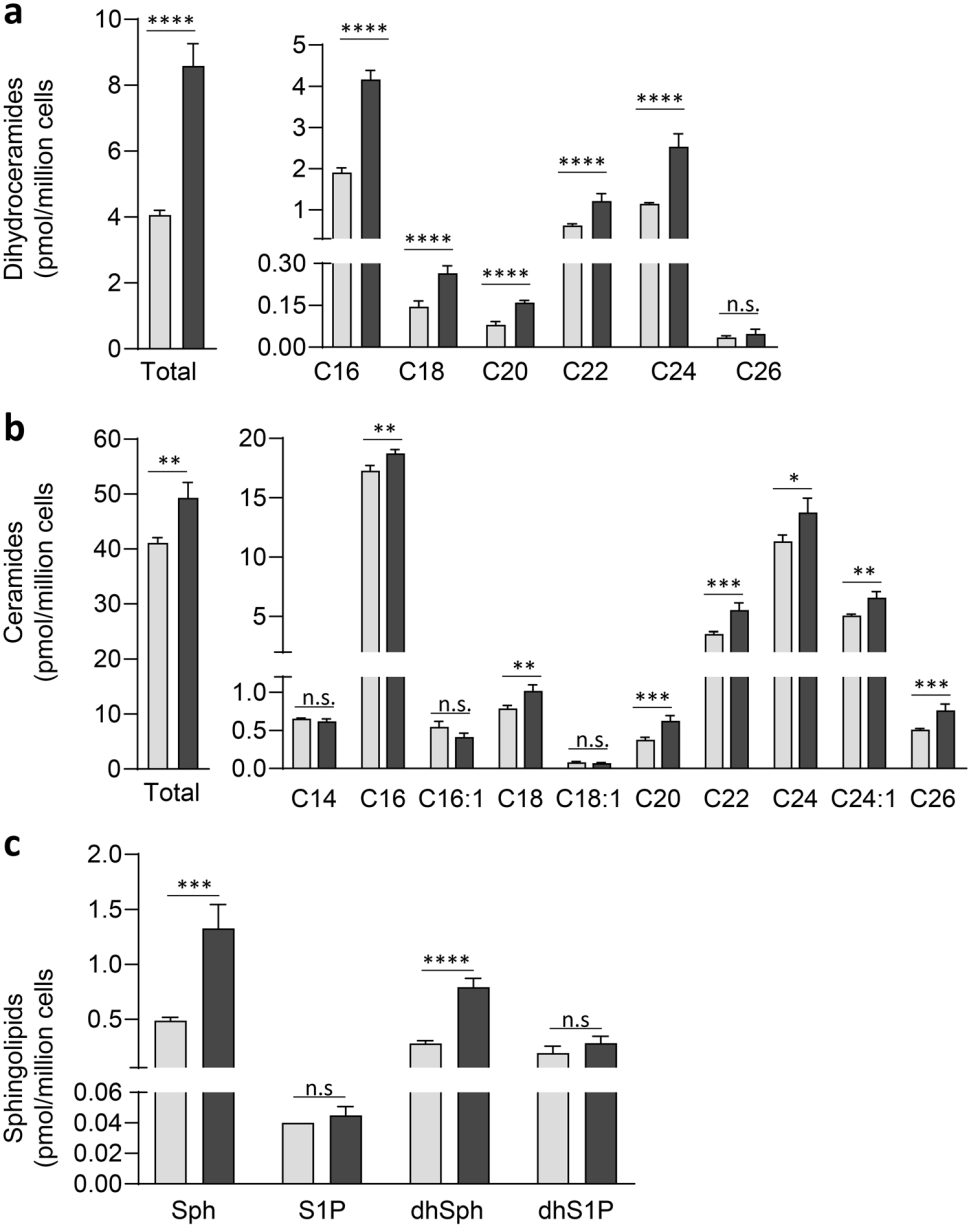
JTE-013 induces altered sphingolipid levels in MV411 cells. MV411 cells were treated with vehicle (DMSO, 0.1% final) control (Grey) or 10 μM JTE-013 (Black) for 6 h and analysed by LC–MS. (**a**) Quantitation of total dihydroceramides (LHS) and individual dihydroceramide species (RHS). (**b**) Quantitation of total ceramides (LHS) and individual ceramide species (RHS). Only the more abundant dhCer and Cer species are shown, with quantitation of the full list of species shown in Supplemental Table S1. (**c**) Quantitation of sphingosine, S1P, dihydrosphingosine and dihydroS1P. All results are presented as pmol/million cells, mean ± SEM (n = 4 independent treatments). Statistical significance was determined by Student’s t-test (*p* value *< 0.05, **< 0.01 ***< 0.001, ****< 0.0001, n.s. not significant).

We have previously demonstrated that SK1 inhibition induced Mcl-1 degradation and cell death in AML cells, and that this could be recapitulated by the S1P_2_ antagonist JTE-013, implicating the SK1-S1P-S1P_2_ axis in controlling Mcl-1 protein levels^13^. Since JTE-013 has been suggested to have other, albeit undefined cellular targets^10,15,18^, especially at the 10 µM concentration we had previously employed, this led us to investigate the effect of JTE-013 on sphingolipid metabolism. In order to probe the effects of JTE-013 on the levels of cellular sphingolipids we treated MV411 AML cells with 10 µM JTE-013, or vehicle (DMSO) control for 6 h, a time we previously demonstrated could result in Mcl-1 degradation^13^, and then subjected the cells to LC–MS analysis. Surprisingly, JTE-013 treatment caused significant changes in the abundance of various sphingolipids (Fig. 1). Most notably, JTE-013 treatment caused a significant increase in total dihydroceramide (dhCer) levels in the cells, with this largely resulting from significant increases in the most abundant dhCers: C16-dhCer (2.2-fold), C22-dhCer (twofold) and C24-dhCer (2.2 -fold) (Fig. 1A and Supplemental Table S1). JTE-013-treated cells also had significant increases in total ceramide (Cer), again largely resulting from increases in the most abundant ceramides: C16-Cer (1.1-fold), C22-Cer (1.6-fold) and C24-Cer (1.2-fold) (Fig. 1B and Supplemental Table S1). Cellular sphingosine and dihydrosphingosine were also increased in response to JTE-013, 2.7-fold and 2.8-fold, respectively. However, JTE-013 treatment did not significantly alter the cellular levels of S1P, dihydroS1P (Fig. 1C), sphingomyelins or dihydrosphingomyelins (Supplemental Fig. S1).

### JTE-013 inhibits dihydroceramide desaturase 1

**Figure 2:**
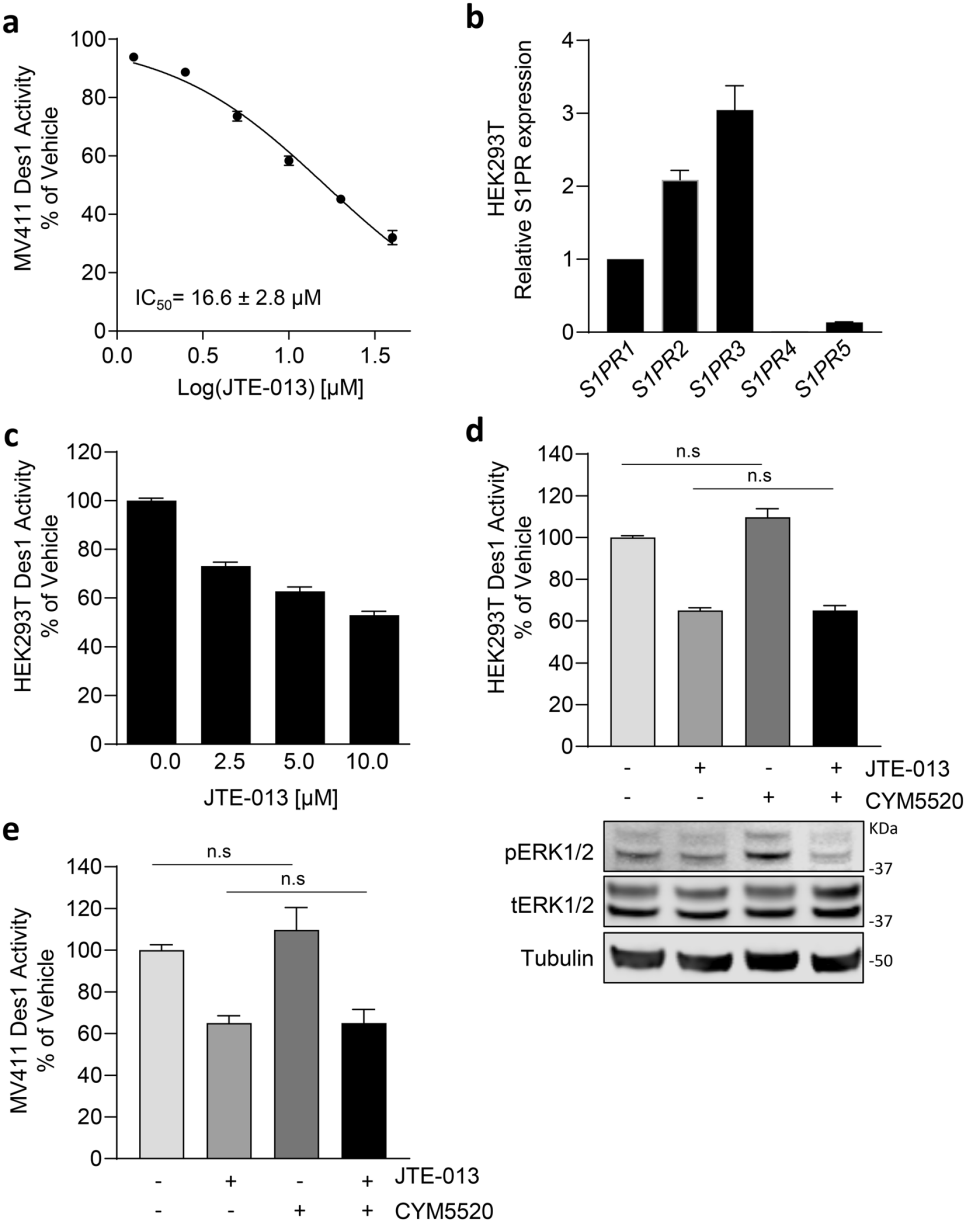
JTE-013 inhibits Des1 activity independently of S1P_2_ receptor. (**a**) MV411 cells were labelled with dhCer and treated with either vehicle control (DMSO, 0.1% final) or varying doses of JTE-013 (40, 20, 10, 5, 2.5, 1.25 µM) in 0.5% FBS/RPMI media for 3 h prior to analysis by HPLC. IC_50_ data was determined using non-linear regression. (**b**) QPCR analysis of HEK293T cells to detect expression of S1P receptor mRNA. Results are presented relative to S1P_1_ and are representative of triplicate measurements in two independent experiments, mean ± SEM. (**c**) HEK293T cells were labelled with NBD-C6-dhCer and treated with either vehicle control (DMSO, 0.1% final) or varying doses of JTE-013 (10, 5, 2.5, 1.25 µM) in 0.5% FBS/DMEM media for 2 h prior to analysis by HPLC. (**d**) To test if S1P_2_ agonism could rescue JTE-013-induced Des1 inhibition, HEK293T cells were labelled with NBD-C6-dhCer and treated with either vehicle control (DMSO, 0.2% final), 10 µM JTE-013, 10 µM S1P_2_ agonist CYM5520 or both in 0.5% FBS/DMEM media for 2 h prior to analysis by HPLC. To confirm agonism of S1P_2_, HEK93T cell lysates from the treatment groups were subjected to SDS-PAGE and western blotting to detect phospho-ERK1/2 (upper panel), total ERK (middle panel) with tubulin as a loading control (bottom panel). Full immunoblots are shown in Supplemental Fig. S2. (**e**) MV411 cells were labelled with NBD-C6-dhCer and treated with either vehicle control (DMSO, 0.2% final), 10 µM JTE-013, 10 µM S1P_2_ agonist CYM5520 or both in 0.5% FBS/DMEM media for 3 h prior to analysis by HPLC. All Des1 data is displayed as a percentage Des1 activity relative to vehicle control; mean ± SEM (n = 3). Statistical significance was determined by Student’s t-test; (*p* value *< 0.05, **< 0.01), n.s. not significant.

The most substantial fold-change in sphingolipids induced by JTE-013 were dihydroceramides, suggesting the potential for additional inhibition of dihydroceramide desaturase 1 (Des1) by JTE-013. To examine this hypothesis, we employed an established Des1 assay using cells labelled with fluorescent NBD-C6-dhCer and followed the conversion of this substrate to NBD-C6-Cer using HPLC analysis. Using this assay in intact MV411 cells we observed that JTE-013 caused a dose-dependent inhibition of ceramide formation, with an IC_50_ of 16.6 ± 2.8 µM, indicating that JTE-013 inhibits Des1 activity (Fig. 2A).

Since JTE-013 is known to antagonise S1P_2_ and S1P_4_^10,15^, which are both expressed at the mRNA level in MV411 cells^13^, we next examined if the observed inhibition of Des1 by JTE-013 in these cells was downstream of S1P_2/4_ antagonism. Since human embryonic kidney HEK293T cells lack S1P_4_ (Fig. 2B) we assessed the effect of JTE-013 on Des1 activity in these cells. We observed very similar Des1 inhibition by JTE-013 to that seen in MV411 cells (Fig. 2C), suggesting antagonism of S1P_4_ was not involved in Des1 inhibition. In order to rule out the potential for this Des1 inhibition to be a downstream effect of S1P_2_ antagonism we tested the ability for the S1P_2_ agonist CYM5520 to rescue the Des1 inhibition by JTE-013 in HEK293T cells. Treatment of HEK293T cells with CYM5520 induced a marked increase in phospho-ERK1/2 (a downstream marker of S1P_2_ activation). As expected, co-treatment with JTE-013 was able to block this phospho-ERK1/2 activation (Fig. 2D). Of note, agonism of S1P_2_ by CYM5520 had no significant effect on Des1 activity in HEK293T and failed to rescue Des1 inhibition by JTE-013 (Fig. 2D). In addition, CYM5520 also failed to rescue Des1 inhibition by JTE-013 in MV411 (Fig. 2E), suggesting that the Des1 inhibition by JTE-013 was independent of its antagonistic effects on S1P_2_.

### JTE-013 directly inhibits Des1 in a dihydroceramide-competitive manner

**Figure 3:**
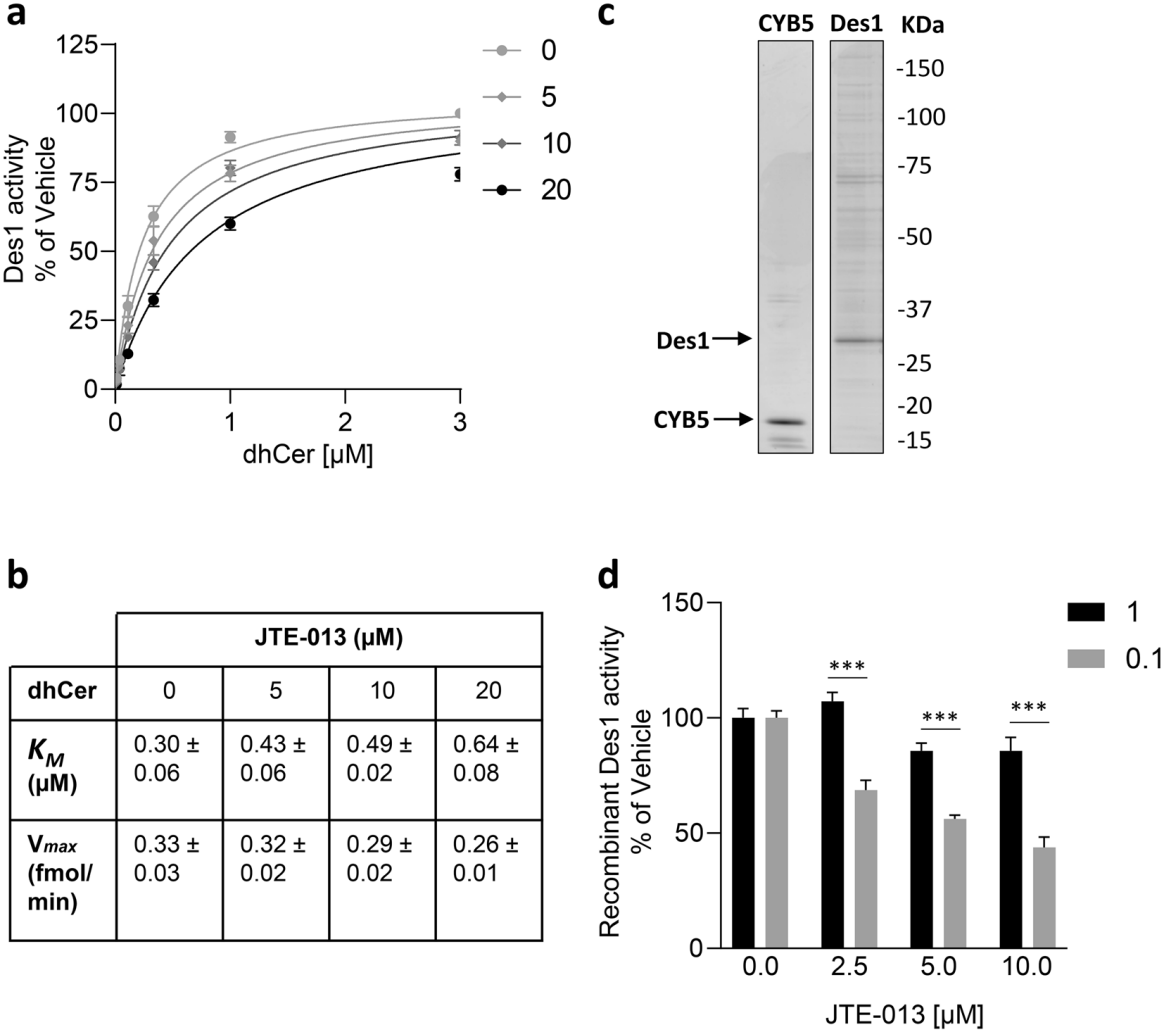
JTE-013 directly inhibits Des1 in a dihydroceramide-competitive manner. (**a**) Lysates from Des1-FLAG expressing HEK293T cells were assayed with varying doses of NBD-dhCer-C6 (3, 1, 0.3, 0.1, 0.03, 0.01 µM, solubilized in fatty acid-free BSA) and NADPH (1 mM final) with vehicle (DMSO, 0.7% final) or 5, 10 or 20 µM JTE-013 and production of NBD-Cer-C6 was quantified by HPLC. (**b**) Curve fitting analysis by non-linear regression was performed to determine the *K*_*M*_, *V*_*max*_ and *K*_i_ = 9.2 ± 0.8 µM. (**c**) Des1-FLAG was overexpressed in HEK293T cells for 24 h, purified using FLAG-agarose beads and eluted with FLAG peptide. Recombinant CYB5 (Sigma) and purified Des1-FLAG were separated by SDS-PAGE and detected by SYPRO-RUBY staining. Full SDS-PAGE gels are shown in Supplemental Fig. S3. (**d**) Purified Des1-FLAG was assayed using 1 µM (Black bars) or 0.1 µM (Grey bars) doses of NBD-dhCer-C6 (solubilized in fatty acid-free BSA) with vehicle (DMSO, 0.7% final) or 2.5, 5, or 10 µM JTE-013. Inhibition by JTE-013 was rescued by higher doses of dhCer indicating competitive mode of inhibition. All results are presented as percent of vehicle control; mean ± SEM (n = 3). Statistical significance was determined by Student’s t-test (*p* value ***< 0.001).

Since the data from our intact cell-based Des1 assays suggested that JTE-013 inhibited Des1 we next investigated the mode of inhibition by JTE-013. In order to tightly control the levels of dhCer substrate we employed a microsomal Des1 assay using sucrose-solubilized membranes from HEK293T cells overexpressing Des1. Assaying Des1 in the presence of varying concentrations of dhCer substrate with multiple JTE-013 concentrations revealed an apparent competitive mode of inhibition, where the *K*_*M*_ for dhCer increased without a change in the V_max_ (Fig. 3A,B). Non-linear regression analysis determined a *K*_i_ of 9.2 ± 0.8 µM with respect to dhCer. To verify this mode of inhibition we generated purified recombinant Des1 and used it together with purified cytochrome B5 (Fig. 3C), its cofactor required for catalytic activity, to test JTE-013 inhibition with high dhCer (1 µM) and low dhCer (0.1 µM) concentrations (from Fig. 3B the Des1 *K*_M_ for dhCer was determined to be 0.3 µM). Consistent with a competitive mode of inhibition, JTE-013 dose-dependently inhibited Des1 at low (0.1 µM) dhCer but the inhibition was significantly less when assayed in the presence of high (1 µM) dhCer (Fig. 3D).

### JTE-013 inhibits sphingosine kinases but not S1P lyase

**Figure 4:**
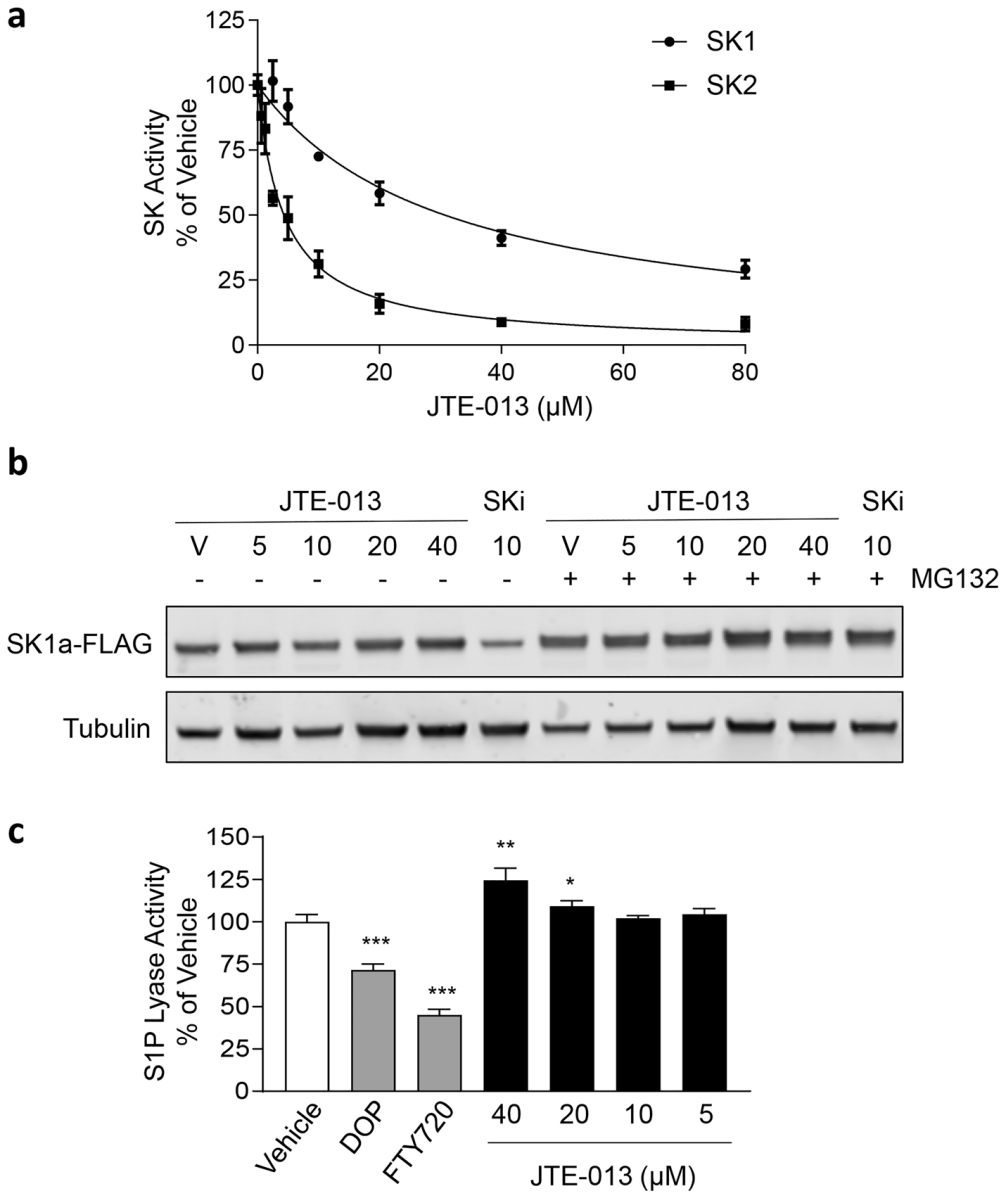
S1P_2_ antagonist JTE-013 inhibits sphingosine kinases but not S1P lyase. (**a**) Purified recombinant human sphingosine kinases 1 and 2 were assayed with vehicle (DMSO), or varying doses of JTE-013. Values are shown as % activity compared to vehicle control. Lines indicate non-linear regression curve fitting (GraphPad Prism). Results represent mean ± SEM from three independent experiments performed in duplicate. (**b**) Western blots show the effect of varying concentrations of JTE-013 (5–40 μM) or 20 μM SKi for 24 h compared to vehicle (V) treatment in the presence or absence of 10 μM MG132 on SK1a-FLAG in HEK293 cells (upper panel). Tubulin was used as a loading control (lower panel). Full immunoblots are shown in Supplemental Fig. S4. Results are representative of 3 independent experiments. (**c**) human S1P lyase was assayed with vehicle (DMSO), 1.25 mM 4-deoxypyridoxine (DOP), 25 µM FTY720, or varying doses of JTE-013. Values are shown as % activity compared to vehicle control and represent mean ± SEM (n = 3). Statistical significance was determined by Student’s t-test (*p* value ***< 0.001, **< 0.01, *< 0.05, compared to vehicle).

While we found JTE-013 to inhibit Des1, the observation that JTE-013 also increased sphingosine and ceramides in MV411 cells suggested the potential for JTE-013 to also inhibit sphingosine kinase activity in these cells. To directly assess the effect of JTE-013 on the SKs we employed in vitro SK assays using purified recombinant human SK1 and SK2. Notably, JTE-013 was found to quite potently inhibit SK2, with an IC_50_ of 4.3 ± 0.5 µM (Fig. 4A). JTE-013 also inhibited SK1, although with less potency, with an IC_50_ of 25.1 ± 2.7 µM (Fig. 4A).

Since a number of SK1 inhibitors, like SKi (also known as SKI-II), cause SK1 degradation^19^, we next tested whether JTE-013 induced degradation of SK1. We employed a doxycycline-inducible system to express low levels of FLAG-tagged SK1 in HEK293 cells. After 16 h of ectopic SK1 expression, doxycycline was removed and cells treated with vehicle or inhibitor, in the absence and presence of proteasomal inhibitor MG132 in order to measure the effect on turnover of the existing SK1 protein^19^. As expected, the SK1/SK2/Des1 inhibitor SKi caused degradation of SK1 that was reversed by co-treatment with the proteasome inhibitor MG132 (Fig. 4B). In contrast, JTE-013 did not induce degradation of SK1, even at concentrations up to 40 µM (Fig. 4B).

While JTE-013 inhibits SK activity, our observations that this did not result in an increase in S1P or dihydroS1P in MV411 cells (Fig. 1C), suggested the potential for JTE-013 to also inhibit S1P lyase and the major route of S1P/dhydroS1P clearance^20^. To assess this, we employed in vitro S1P lyase assays using lysates of HEK293T cells overexpressing human S1P lyase. While known S1P lyase inhibitors 4-deoxypyridoxine^21^ and FTY720^22^ both showed inhibition of S1P lyase activity, JTE-013 showed no such inhibition at concentrations up to 40 µM (Fig. 4C). Indeed, high concentrations of JTE-013 even resulted in slightly enhanced S1P lyase activity in these in vitro assays (Fig. 4C). Thus, the reasons for the observed unaltered S1P levels in response to JTE-013-induced SK inhibition remain unresolved.

### Knockdown of S1P_2_ expression does not alter AML cell survival

**Figure 5:**
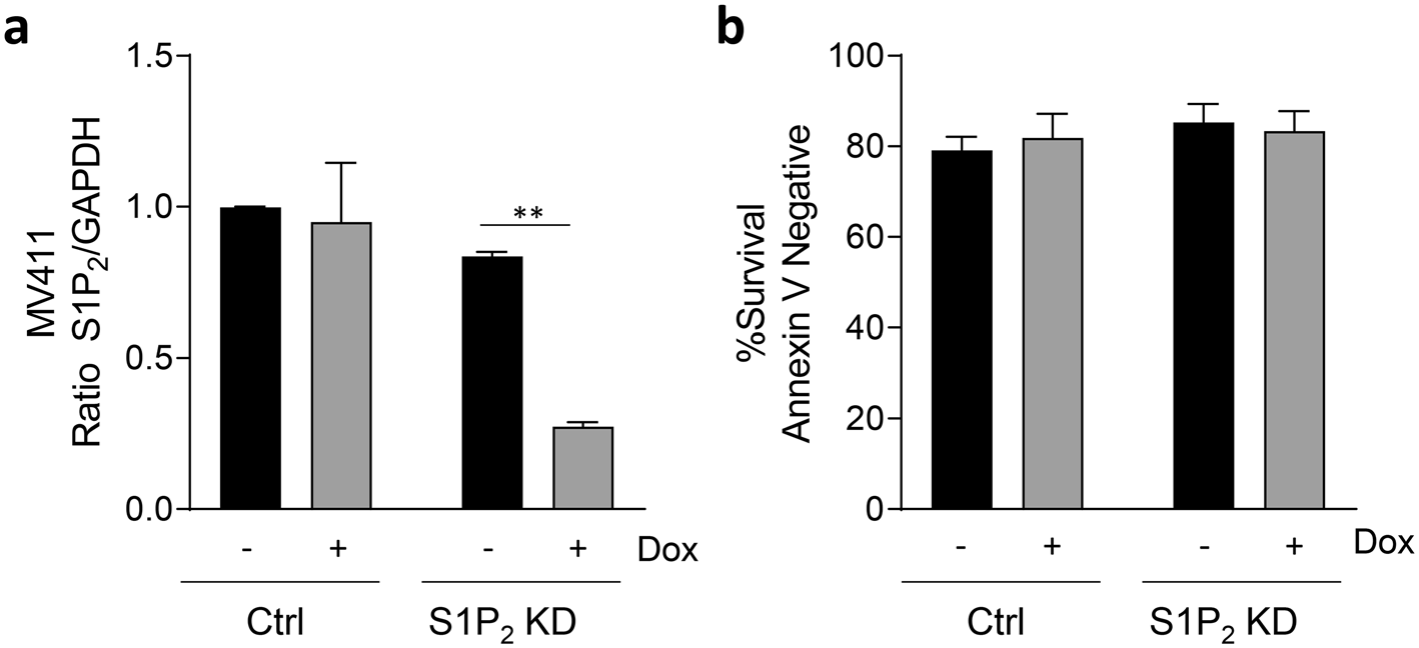
Knockdown of S1P_2_ expression does not alter AML cell survival. Doxycycline-inducible S1P_2_ shRNA and non-targeting control MV411 cell lines were treated with or without doxycycline (1 µg/ml) for 48 h prior to analysis. (**a**) qPCR analysis of S1P_2_ receptor expression relative to GAPDH; mean ± SEM, n = 3. (**b**) analysis of cell survival by Annexin-V staining; mean ± SEM, n = 3. Statistical significance was determined by Student’s t-test (*p* value **< 0.05).

Given our findings that JTE-013 inhibits Des1 and SK1/2 when employed at 10 µM, we re-examined our previous findings, that had suggested targeting S1P_2_ (with JTE-013) induced Mcl-1 degradation and cell death in AML cells^13^. Employing MV411 cells engineered with doxycycline-inducible expression of S1P_2_ shRNA, we found that efficient S1P_2_ knockdown (> 70%; Fig. 5A) had no effect on the viability of these cells (Fig. 5B). Thus, in contrast to our previous studies, this indicates that it is unlikely that S1P_2_ has a pro-survival role in AML cells, and emphasizes the need for caution in interpreting findings from the use of JTE-013, especially at concentrations around 10 µM.

## Discussion

JTE-013 has been widely employed to interrogate the role of S1P_2_ in various biological processes. While JTE-013 shows selectivity to S1P_2/4_ over other S1P receptors, the specificity of this molecule has not been very well examined. In this study we assessed the impact of JTE-013 on sphingolipid metabolism and found broad effects that appear to result from its inhibition of both Des1 and the SKs. Previous studies have questioned the specificity of JTE-013, particularly when used at higher concentrations, such as 10 µM^17^, primarily due to observations that it blocked S1P signalling in S1P_2_ knockout mice^15^ or had effects on other cells lacking S1P_2_ expression^16^. The “off-targets” of JTE-013 have, however, not been definitively identified prior to the current study.

Dual Des1/SK inhibition appears to be a common feature in a number of small molecule inhibitors, having been previously reported for SKi^23,24^ and ABC294640^24,25^. Intriguingly, while JTE-013 shows common features to SKi in the ability to inhibit both SK1 and SK2 as well as Des1, it also displays differences in its mode of inhibition. JTE-013 inhibits Des1 in a competitive manner with respect to dhCer while SKi is a non-competitive inhibitor of Des1^23^. In addition, SKi and ABC294640 can both bind to the sphingosine binding pocket of SK1^26,27^ and SK2^28^, respectively, but both can also induce the degradation of SK1^19,23,24^ whilst JTE-013 did not induce the degradation of SK1. Together, the observed overlapping functions of these inhibitors highlights the difficultly in developing selective agents for the sphingolipid pathway enzymes and receptors and the importance of rigorous cross-screening during target validation for small-molecule inhibitors.

Our findings call into question many of the previous studies using JTE-013 at micromolar concentrations on cultured cells in vitro, and those without knockdown/knockout validation. This includes our own previous studies where we had employed JTE-013 to implicate S1P_2_ in a role in AML cell survival through the stabilization of the pro-survival Mcl-1 protein^13^. Indeed, our previous data demonstrated that 5–10 µM of JTE-013 was required to induce loss of Mcl-1 and AML cell death, and phenocopy the effects of targeting SK1^13^. In light of our current findings, with the knockdown of S1P_2_ having no impact on AML cell viability (Fig. 5B), it is most likely that our previous findings with JTE-013 were due to SK1 inhibition and altered sphingolipid metabolism, rather than due to antagonism of S1P_2_.

While our findings emphasize caution in the use of JTE-013, it should be noted that with the paucity of selective S1P_2_ antagonists there currently remains a place for JTE-013 as a tool to interrogate the roles of S1P_2_. Consistent with previous proposals^17^, our findings would suggest that use of JTE-013 at concentrations of 1 µM and below should infer selectivity for S1P_2_ over its effects on Des1 and the sphingosine kinases, with the caveat that this will depend on the level of endogenous dihydroceramides and sphingosine that are present to compete for JTE-013. The lack of availability of *bona fide* S1P_2_ selective antagonists requires the use of several controls to uncover any roles that S1P_2_ may have, and therefore there is a need for reliable tool compounds that selectively target the S1P receptors.

In summary, there has been robust discussion about the selectivity and specificity of JTE-013 and its reliability as a S1P_2_ antagonist in cellular studies^17,18^. Our current study has uncovered three “off-targets” of JTE-013 in the sphingolipid signaling pathway, with Des1, SK1 and SK2 found to be inhibited by JTE-013 at low micromolar concentrations. Our findings highlight the difficulty in developing selective agents that target the lipid binding pockets of sphingolipid enzymes and receptors and raises a note of caution for using JTE-013 at concentrations above 1 µM in cell-based studies for mechanistic studies. However, with respect to the therapeutic effects observed with JTE-013^9,29–31^, the changes in sphingolipids, may provide an opportunity to partner JTE-013 with other drugs currently used in the clinic, particularly in a cancer setting. As ceramide accumulation is an important event for chemotherapy efficacy and that SK1 inhibitors synergise with chemotherapy^13,32^, it is possible that JTE-013 may broadly synergise with anti-neoplastic drugs, as we have previously shown with venetoclax in AML cells^13^. In addition, a broad targeting agent such as JTE-013 that hits multiple arms of the sphingolipid pathway may prove favorable for therapeutic use in a range of other diseases, where sphingolipid metabolism appears important^33–38^. Such a paradigm already has precedent with the clinical development of FTY720 and ABC294640, which are known to have multiple targets in the sphingolipid pathway^25,39^.

## Methods

### Materials

JTE-013, C6 NBD dihydroceramide (NBD-C6-dhCer) and (2S,3R)-2-ammonio-3-hydroxy-5-((2-oxo-2H-chromen-7-yl)oxy)pentyl hydrogen phosphate were from Cayman Chemical (Ann Arbor, MI). SKi (4-[[4-(4-chlorophenyl)-2-thiazolyl]amino]phenol)^40^, dithiothreitol (DTT), fatty acid-free bovine serum albumin (BSA), human cytochrome B5 (CYB5, Sigma #C1427, E.C.1.6.2.2), FLAG-agarose beads, FLAG peptide, dodecyl maltoside (DDM), SYPRO-RUBY, Complete© EDTA-free protease inhibitors (CPI), Na_3_VO_4,_ pyridoxal 5′ phosphate, trifluoracetic acid and acetonitrile were from Merck (Frenchs Forest, NSW, Australia). MG132 was purchased from Selleck Chem (Houston, TX). HPLC columns and vials were purchased from Phenomenex (Torrence, CA, USA).

### Cell lines and constructs

Generation of Human Embryonic Kidney (HEK)293 cells with doxycycline-inducible expression of FLAG-tagged SK1 was described previously^41^. FlpIn SK1-FLAG HEK293 cells and HEK293T cells (ATCC) were maintained in DMEM supplemented with 10% fetal bovine Serum (FBS; HyClone ThermoFisher Scientific) and 1% penicillin–streptomycin (Gibco). MV411 AML cells (ATCC; authenticated by short tandem repeat profiling) were maintained in RPMI supplemented with 10% FBS (non-heat inactivated) and 1% penicillin–streptomycin (Gibco). Human Des1 (E.C. 1.14.19.17, *DEGS1*) cDNA (Genbank accession number NM_003676) was amplified from human bone marrow cDNA and FLAG epitope-tagged at the 3′ end by polymerase chain reaction (PCR) with Q5 (New England Biolabs, Ipswich, MA) and oligonucleotide primers 5′-TAGAATTCGCCACCATGGGGAGCCGCGTC-3′ and 5′-TAGGATCCTCACTTGTCATCGTCGTCCTTGTAGTCCTCCAGCACCATCTCTCCT-3′. The PCR product was treated with T4 polynucleotide kinase and then digested with EcoRI. pcDNA3 (Invitrogen) was digested with EcoRI and EcoRV prior to ligation with the PCR product to generate pcDNA3-Des1-FLAG. Sequencing verified the integrity of the cDNA.

To generate doxycycline-inducible S1P_2_ shRNA contructs the pTRIPz lentiviral vector was modified with the addition of a poly-linker as described previously^13^. shRNA target sequences from Fellmann et al.^42^, *S1PR2* (5′ TGCTGTTGACAGTGAGCGCAAGGCACTGACTAGTCACATATAGTGAAGCCACAGATGTATATGTGACTAGTCAGTGCCTTATGCCTACTGCCTCGGA) and Renilla luciferase 713 negative control (5′ TGCTGTTGACAGTGAGCGCAGGAATTATAATGCTTATCTATAGTGAAGCCACAGATGTATAGATAAGCATTATAATTCCTATGCCTACTGCCTCGGA). shRNA oligonucleotides were amplified using EcoRI MirE primers (5′ TAGAATTCTGCACTTCTTAACCCAACAGAAGGCTCGAGAAGGTATATTGCTGTTGACAGTGAGCG, 3′ TCTCGAATTCTAGCCCCTTGAAGTCCGAG-GCATAGGC). PCR products were digested with EcoRI and cloned into pTRIPZ. To generate lentivirus, HEK293T cells were co-transfected with this vector (or empty pTRIPZ) and pLP1 (gag/pol), pLP2 (rev), pTAT and pVSVG vectors using Lipofectamine 2000 (Thermo Fisher Scientific) according to the manufacturer’s instructions. Two days after transfection the media was changed from DMEM (10% FBS) to RPMI (10% FBS) and incubated for a further two days. The viral supernatant was then added to 5 × 10^6^ MV411 cells containing 4 µg/ml polybrene and incubated for an additional 72 h prior to selection with 1 µg/ml of puromycin. Doxycycline induced RFP expression confirmed transduction efficiencies of > 80%.

Human S1P lyase cDNA (SGPL1, Genbank Accession number NM_003901) was amplified from placenta cDNA and FLAG epitope-tagged at the 3′ end by PCR with oligonucleotide primers 5′-TATATAGAATTCGCCACCATGCCTAGCACAGACCTTCT-3′ and 5′-TATATAGAATTCACTTGTCATCGTCGTCCTTGTAGTCGTGGGGTTTTGGAGAACCAT-3′. The PCR product was digested with EcoRI and cloned into pcDNA3 (Invitrogen) for expression in mammalian cells. Sequencing verified the orientation and integrity of the cDNA.

### Quantitative RT-PCR

Analysis of HEK293T gene expression of *S1PR1-5* was performed by quantitative reverse transcriptase PCR (qPCR) as previously described^13^ using 5 × 10^6^ HEK293T cells. Expression of the *S1PR1-5* was analysed using the Rotor-Gene Q Series software (Qiagen) using the comparative quantitation method with HEK293T amplified *S1PR1* used as the calibrator. Gene expression of *S1PR2* in control and S1P_2_ shRNA MV411 cell lines was analysed as above, normalized to GADPH and analysed using the Rotor-Gene Q Series software (Qiagen) using the comparative quantitation method with MV411 amplified *S1PR2* used as the calibrator.

### Intact cell assay for Des1 activity

For the non-adherent MV411 cells, Des1 assays were performed as previously described^24^. For assays using adherent HEK293T, the cells were harvested by trypsin and counted prior to use in the assay. Briefly, intact cells (at 1 × 10^6^ cells/mL) were labeled with NBD-C6-dhCer (5 µM) in serum-free media and incubated on ice for 30 min. Cells were then pelleted and washed into fresh media (with 0.5% FBS) and dispensed into 24-well plates containing inhibitor/vehicle treatments (400 µL total) and incubated for 2 h at 37 °C and 5% CO_2_. The cells were then harvested and pelleted via centrifugation at 500×*g* and resuspended in 100 µL H_2_O. The samples were sonicated for 30 s in a bath sonicator (Diagenode) followed by the addition of 900µL cold methanol. Immediately before analysis, the samples were centrifuged for 3 min at 17,000×*g* and transferred to glass HPLC vials (Phenomenex). Samples (50 μL) were analysed on a Waters HPLC coupled to a fluorescence detector using a 30 cm C18 reverse-phase column (Phenomonex) eluted with 1 mL/min 20% H_2_O and 80% acetonitrile with 0.1% trifluoroacetic acid. NBD-labelled substrate and product were quantitated with excitation and emission wavelengths of 465 nm and 530 nm, respectively. Des1 inhibition was calculated relative to vehicle as percentage peak area under the curve.

### Des1 assays using cell lysates

Cell lysate preparations of FLAG-tagged Des1 were used in the kinetic assays. HEK293T cells were transfected with pcDNA3-Des1FLAG (10 μg) using Lipofectamine 2000 (ThermoFisher Scientific, 40 μL) according to the manufacturer’s instructions. At 24 h post transfection, HEK293T cells were harvested, pelleted and snap frozen in liquid nitrogen. Pellets were thawed and resuspended in 200 μL of Buffer A (50 mM Tris–HCl buffer, pH 7.4, 50 mM sucrose and CPI). Cells were lysed by 10 passages through a 26G needle and placed on ice for 10 min. The protein concentration was determined by Bradford assay (BioRad) and 30 µg of lysate was used per assay. Stock solutions of 30 µM NBD-C6-dhCer were prepared in 50 mM Tris–HCl buffer (pH 7.4) containing 10% (w/v) fatty acid-free BSA and solubilized by sonication on ice (Diagenode Bioruptor). Assays were performed with 0.01 to 3 µM NBD-C6-dhCer substrate (with a constant concentration of fatty acid-free BSA (2.6% final), in 50 mM Tris–HCl buffer (pH 7.2) containing 1 mM NADPH. After 30 min at 37 °C, the reaction was stopped by the addition of cold methanol (400 μL) to each sample and samples were centrifuged at 17,000×*g* for 3 min at room temperature. Lipid extracts were transferred to glass HPLC vials and analysed using area under the curve. Curve fitting was performed using non-linear regression in GraphPad (Prism v8.3.0).

### Purification of recombinant Des1

HEK293T cells were transfected with pcDNA3-Des1FLAG and collected 24 h later, resuspended in 100 mM Tris–HCl buffer (pH 7.0) containing 5% glycerol and CPI), lysed by 20 passages through a 26G needle, and then centrifuged at 100,000×*g* for 1 h at 4 °C (Beckman) to pellet the membrane fraction. The supernatant was discarded, and the pellet was gently resuspended in 100 mM Tris–HCl buffer (pH 7.0) with 5% glycerol, 800 mM NaCl, 1% (w/v) DDM and CPI. Samples were incubated with gentle mixing for 30 min at 4 °C to solubilize the membranes, and insoluble products were pelleted by centrifugation at 100,000×*g* for 1 h at 4 °C. The solubilized membranes were collected in the supernatant, diluted 1:2 in 100 mM Tris–HCl buffer (pH 7.0) with 5% glycerol, 400 mM NaCl and 0.5% DDM and mixed with FLAG-agarose beads for 1 h at 4 °C. The beads were washed three times with 100 mM Tris–HCl pH 7.0 with 5% glycerol, 200 mM NaCl and 0.25% DDM and then Des1-FLAG was eluted for 30 min at 4 °C with 0.2 µg/µL FLAG peptide in 100 mM Tris–HCl buffer (pH 7.0) containing 200 mM NaCl and 0.1% DDM. Purity of samples was assessed by SDS-PAGE and SYPRO-RUBY staining. Protein was used fresh for all downstream assays.

### Recombinant Des1 assay

Purified Des1-FLAG protein was assayed with NBD-C6-dhCer in 1% fatty acid-free BSA, 1 mM NADPH, 50 µM (NH_4_)_2_Fe(SO_4_)_2_ (prepared with 3 × molar excess ascorbic acid) and recombinant cytochrome B5 (CYB5) in 50 mM Tris–HCl buffer (pH 7.2) with 1 mM DTT. The reaction was incubated at 37 °C for 30 min, and then terminated by the addition of methanol and centrifugation at 17,000×*g* for 5 min. Lipid extracts were transferred to glass HPLC vials and analysed as above.

### S1P lyase assay

S1P lyase activity was assayed essentially as described previously^43^. Briefly, HEK293T cells overexpressing FLAG-tagged human S1P lyase were lysed by sonication (Diagenode Bioruptor; 5 × 30 s cycles) on ice into 50 mM HEPES buffer (pH 7.5) containing 25 µM Na_3_VO_4_ and complete protease inhibitors (Roche). Whole cell lysates (50 µg protein) were incubated with vehicle (DMSO) or inhibitor in 50 mM HEPES buffer (pH 7.5) containing 25 µM Na_3_VO_4_ for 30 min. Pyridoxal 5’ phosphate (20 µM) and the substrate (2S,3R)-2-ammonio-3-hydroxy-5-((2-oxo-2H-chromen-7-yl)oxy)pentyl hydrogen phosphate (50 µM) were then added and the reaction mix incubated at 37 °C for 6 h. The mixture was then transferred to 96-well black plates and fluorescence (ex/em: 355/460 nm) measured on a FLUOstar Omega spectrophotometer (BMG Labtech).

### Mass spectrometry sphingolipidomics

MV411 cells (1.5 × 10^7^) were seeded at 1 × 10^6^ per mL and treated with DMSO (0.1% final) or 10 µM JTE-013 for 6 h. Cells were pelleted by centrifugation, washed in PBS and snap frozen. The cell pellets were suspended in 1 mL of chilled PBS and centrifuged at 2000×*g* for 5 min at 4 °C. The supernatant was discarded and the remaining solid was resuspended in 450 µL of extraction mix [chloroform: methanol: H_2_O, 2:6:1 (v/v)]. Odd chain lipid standards mix was added to give final concentrations of 241 nM sphingosine (d17:1), 250 nM dihydosphingosine (d17:0), 253 nM S1P (d17:1), 235 nM dihydrosphingosine 1-phosphate (d17:0), 250 nM sphingomyelin (d18:1/17:0), and 450 nM C17 ceramide (d18:1/17:0). The samples were frozen/thawed three times, then centrifuged at 14,800×*g* for 5 min at 4 °C. 400 µL of supernatant was then transferred to new tubes, with some remaining sample combined to make a pooled QC sample. To reconstitute, the solvent was evaporated to dryness keeping the samples in a centrifugal evaporator at 55 °C for 50 min. Dried extracts were frozen at – 80 °C until LC–MS analysis was performed. On the day of analysis, the samples were dissolved in 180 µL of butanol: methanol (v/v 1:1) mixture and 20 µL of water. The samples were vortexed on a rotary vortex for 10 min and sonicated in a sonicator bath for 1 h keeping the temperature below 25 °C. The samples were centrifuged at 14,800×*g* for 10 min at 20 °C and transferred to LC–MS vials.

LC–MS data was acquired on a Q-Exactive Orbitrap mass spectrometer (Thermo Fisher Scientific) coupled with high-performance liquid chromatography (HPLC) system Dionex Ultimate® 3000 RS (Thermo Fisher Scientific). Chromatographic separation was performed on a C8 Ascentis Express® column (2.7 µm, 2.1 × 100 mm, Supelco, Merck). The mobile phase (A) was 40% isopropanol, 8 mM ammonium formate, 2 mM formic acid and (B) 98% isopropanol 8 mM, ammonium formate, 2 mM formic acid. Needle wash solution was 50% isopropanol. The gradient program started at 0% B and was increased stepwise to 20% B over 1.5 min, to 28% B over 5.5 min, to 35% B over 1 min, to 65% B over 16 min and to 100% B over 1 min. Wash at 100% B was continued for 2 min before decreasing to 0%B over the next 2 min followed by equilibration at 0% B for 1 min. The flow rate was 0.2 mL/min and column compartment temperature 40ºC. The total run time was 30 min with an injection volume of 10 µL. The mass spectrometer operated in full scan mode with positive and negative polarity switching at 70,000 resolution at 200 m/z with detection range of 140 to 1300 m/z. Electro-spray ionization source (HESI) was set to 3.5 kV voltage for positive mode and 3.5 kV for negative mode, sheath gas was set to 34, aux gas to 13 and sweep gas to 1 arbitrary units, capillary temperature 250 °C, probe heater temperature 190 °C. The samples were analyzed as a single batch to avoid batch-to-batch variation and randomized to account for LC–MS system drift over time.

Using an internal standard approach, 58 sphingolipid species were identified based on accurate mass and retention time, with the majority of sphingolipids detected with excellent precision (RSD < 10%). Quantitation was performed by semi-automated peak integration using Tracefinder 3.2 (Thermo Fisher) with manual verification. The sample concentrations were calculated based on the ratio of peak area of each identified lipid component over the area of the corresponding internal standard (C17 ceramide was used as internal standard for all ceramides). Concentrations were then converted to pmol/1 × 10^6^ cells by dividing calculated concentration by cell number. For simplicity of nomenclature, ceramides with no double bonds were defined as dihydroceramides, those with one double bond defined as ceramides, and those with two double bonds defined as ceramides with unsaturated N-linked acyl chains (noting that the latter two classes may contain small contributions from isomeric dihydroceramides with unsaturated N-linked acyl chains).

### Sphingosine kinase assays

Sphingosine kinase assays using purified recombinant SK1 (1 ng) and SK2 (10 ng)^44,45^ were performed with JTE-013 or DMSO using fatty acid-free BSA (0.1%) solubilized-sphingosine (10 µM), ATP (100 µM) and 1 μCi [γ^32^P]ATP in 100 mM Tris/HCl (pH 7.4), containing 150 mM NaCl, 1 mM Na_3_VO_4_, 10 mM NaF (100 µl total reaction volume), incubated for 30 min at 37 °C, as described previously^46^.

### SK1 degradation assays

SK1 degradation assays were performed as described previously^47^. JTE-013 treatments were compared to DMSO vehicle or SKi positive control in the presence or absence of proteasome inhibitor MG132 (10 µM).

### Cell survival assays

Doxycycline-inducible MV411 non-targeting control and S1P_2_ shRNA cell lines were treated with doxycycline (or control) for 48 h prior to analysis. Survival assays were carried out as described previously^13^ using annexin V-FITC (Roche) negativity exclusion using flow cytometry (Gallious, Beckman Coulter).

## Supplementary Information

Supplementary Information.

## Data Availability

All data is presented within the current manuscript or referenced articles.
