# Supplementary material for: The sphingosine 1-phosphate receptor 2/4 antagonist JTE-013 elicits off-target effects on sphingolipid metabolism
Source: Sci Rep. 2022 Jan 10;12:454. doi: 10.1038/s41598-021-04009-w (PMC8748775; doi:10.1038/s41598-021-04009-w)
Supplement: Supplementary file 1 — Supplementary Information. [file 41598_2021_4009_MOESM1_ESM.pdf]

Supplemental Table S1: Sphingolipidomics of MV411 cells treated with 10µM JTE-013 for 6 h.

Name	Assigned Lipid	Vehicle	JTE-013	Fold-Change	p-value
**Ceramides**	**Total**	**41.12 ± 0.97**	**49.32 ± 2.82**	**1.2**	**0.00151**
C14	Cer(d32:1)	0.65 ± 0.01	0.62 ± 0.03	0.9	0.08106
C16-(d17) [a]	Cer(d33:1)	0.13 ± 0.01	0.13 ± 0.02	1.0	>0.999999
C16	Cer(d34:1)	17.28 ± 0.44	18.74 ± 0.33	1.1	0.00178
C16:1	Cer(d34:2)	0.55 ± 0.07	0.41 ± 0.05	0.8	0.02184
C18	Cer(d36:1)	0.79 ± 0.04	1.02 ± 0.08	1.3	0.00235
C18:1	Cer(d36:2)	0.08 ± 0.01	0.07 ± 0.01	0.9	0.09413
C20	Cer(d38:1)	0.38 ± 0.03	0.62 ± 0.06	1.7	0.00063
C22	Cer(d40:1)	3.55 ± 0.18	5.53 ± 0.62	1.6	0.00081
C24-(d17) [a]	Cer(d41:1)	0.51 ± 0.03	0.69 ± 0.07	1.4	0.00263
C24:1-(d17) [a]	Cer(d41:2)	0.29 ± 0.02	0.41 ± 0.04	1.4	0.00214
C24	Cer(d42:1)	11.34 ± 0.53	13.76 ± 1.21	1.2	0.01029
C24:1	Cer(d42:2)	5.07 ± 0.11	6.57 ± 0.53	1.3	0.00143
C26	Cer(d44:1)	0.51 ± 0.01	0.76 ± 0.08	1.5	0.00090
**Dihydroceramides**	**Total**	**4.06 ± 0.14**	**8.58 ± 0.67**	**2.1**	**0.00001**
dhC16	Cer(d34:0)	1.91 ± 0.11	4.16 ± 0.22	2.2	0.00000
dhC18	Cer(d36:0)	0.15 ± 0.02	0.26 ± 0.03	1.8	0.00038
dhC20	Cer(d38:0)	0.08 ± 0.01	0.16 ± 0.01	2.1	0.00003
dhC22	Cer(d40:0)	0.62 ± 23.98	1.21 ± 0.18	2.0	0.00071
dhC24-(d17) [a]	Cer(d41:0)	0.08 ± 0.01	0.14 ± 0.01	1.7	0.00015
dhC24	Cer(d42:0)	1.15 ± 0.02	2.53 ± 0.31	2.2	0.00012
dhC26-(d17) [a]	Cer(d43:0)	0.03 ± 0.01	0.06 ± 0.01	1.8	0.00121

dhC26	Cer(d44:0)	0.04 ± 0	0.05 ±± 0.02	1.3	0.21484
**Sphingomyelins**	**Total**	**221.59 ± 23.98**	**227.32 ± 18.14**	**1.0**	**0.71609**
C12	SM(d30:1)	0.06 ± 0.01	0.07 ±± 0.01	1.2	0.04006
C14	SM(d32:1)	5.45 ± 0.63	5.28 ± 0.38	1.0	0.67407
C16-(d17) [a]	SM(d33:1)	2.98 ± 0.28	3.32 ± 0.29	1.1	0.15124
C16	SM(d34:1)	162.94 ± 16.83	167.81 ± 11.82	1.0	0.65251
C16:1	SM(d34:2)	5.51 ± 0.76	5.82 ± 0.67	1.1	0.55688
C18	SM(d36:1)	3.11 ± 0.36	3.37 ± 0.34	1.1	0.34309
C18:1	SM(d36:2)	0.59 ± 0.04	0.76 ± 0.06	1.3	0.00610
C20	SM(d38:1)	0.78 ± 0.16	0.82 ± 0.15	1.0	0.74863
C22	SM(d40:1)	8.46 ± 1.37	8.33 ± 1.02	1.0	0.88144
C22:1	SM(d40:2)	1.63 ± 0.34	1.51 ± 0.32	0.9	0.63111
C24-(d17) [a]	SM(d41:1)	0.71 ± 0.15	0.71 ± 0.16	1.0	0.98236
C24:1-(d17) [a]	SM(d41:2)	0.93 ± 0.13	0.96 ± 0.14	1.0	0.75012
C24	SM(d42:1)	10.84 ± 1.71	10.61 ± 1.39	1.0	0.84305
C24:1	SM(d42:2)	15.15 ± 2.07	15.61 ± 2.87	1.0	0.80460
C24:2	SM(d42:3)	2.08 ± 0.34	1.96 ± 0.07	0.9	0.50098
C26	SM(d44:1)	0.1 ± 0.02	0.11 ± 0.02	1.1	0.50738
C26:1	SM(d44:2)	0.28 ± 0.05	0.29 ± 0.06	1.0	0.70646
**Dihydrosphingomyelins**	**Total**	**36.96 ± 4.09**	**41.38 ± 3.84**	**1.1**	**0.16542**
dhC14	SM(d32:0)	0.9 ± 0.07	1.06 ± 0.06	1.2	0.01213
dhC16-(d17) [a]	SM(d33:0)	0.08 ± 0.02	0.12 ± 0.02	1.5	0.02547
dhC16	SM(d34:0)	32.12 ± 3.45	36.22 ± 3.42	1.1	0.14247
dhC18	SM(d36:0)	0.98 ± 0.12	1.05 ± 0.09	1.1	0.33484
dhC24	SM(d42:0)	2.88 ± 0.45	2.92 ± 0.43	1.0	0.88947

**dhSph**		0.28 ± 0.02	0.79 ± 0.08	2.8	0.00002
**dhS1P**		0.19 ± 0.06	0.28 ± 0.06	1.5	0.08847
**Sph**		0.49 ± 0.03	1.33 ± 0.21	2.7	0.00025
**S1P**		0.04 ± 0	0.04 ± 0.01	1.1	0.13398

All results are presented as pmol/million cells, mean ± SD (n=4 independent treatments). Statistically significant differences in levels of lipid species (p<0.05; highlighted in grey) was determined using Student's t-test. Lipids were determined by LC-MS and assigned with the assumption of a C18 sphingosine backbone. [a]lipids assumed to have C17 sphingosine backbone. Lipids with 2-3 double bonds have the potential to be sphingadiene backbone or contain multiple double bonds in the acyl chain.

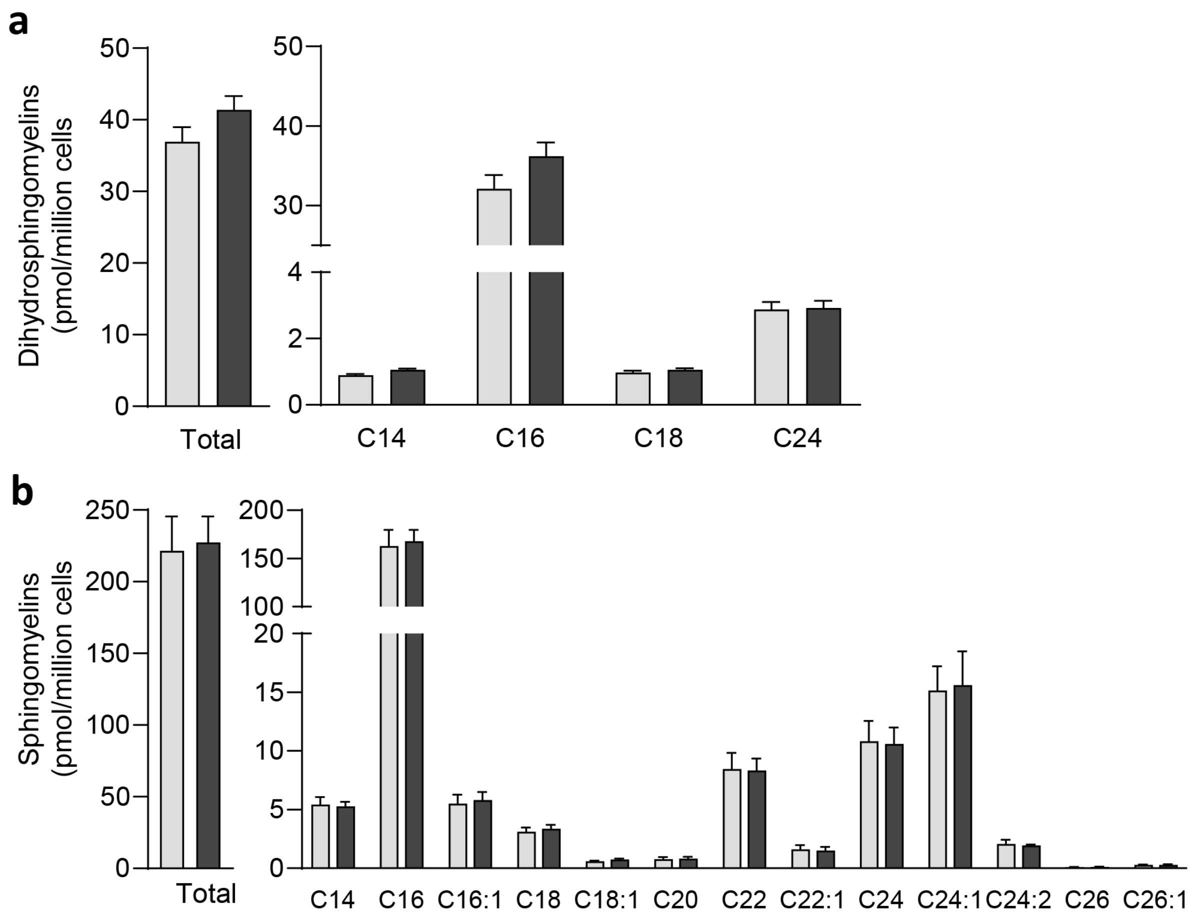

Supplemental Figure S1

MV411 cells were treated with vehicle (DMSO) control (Grey) or 10μM JTE-013 (Black) in quadruplicate for 6 h and analysed by LC-MS. Statistical significance was determined by Student's t-test. All comparisons were determined to be non-significant; *p* value > 0.05). A) Total dihydrosphingomyelins (LHS) and individual dihydrosphingomyelin species (RHS). B) Total sphingomyelins (LHS) and individual sphingomyelin species (RHS). All results are presented as pmol/million cells, mean ± SD (n=4 independent treatments).

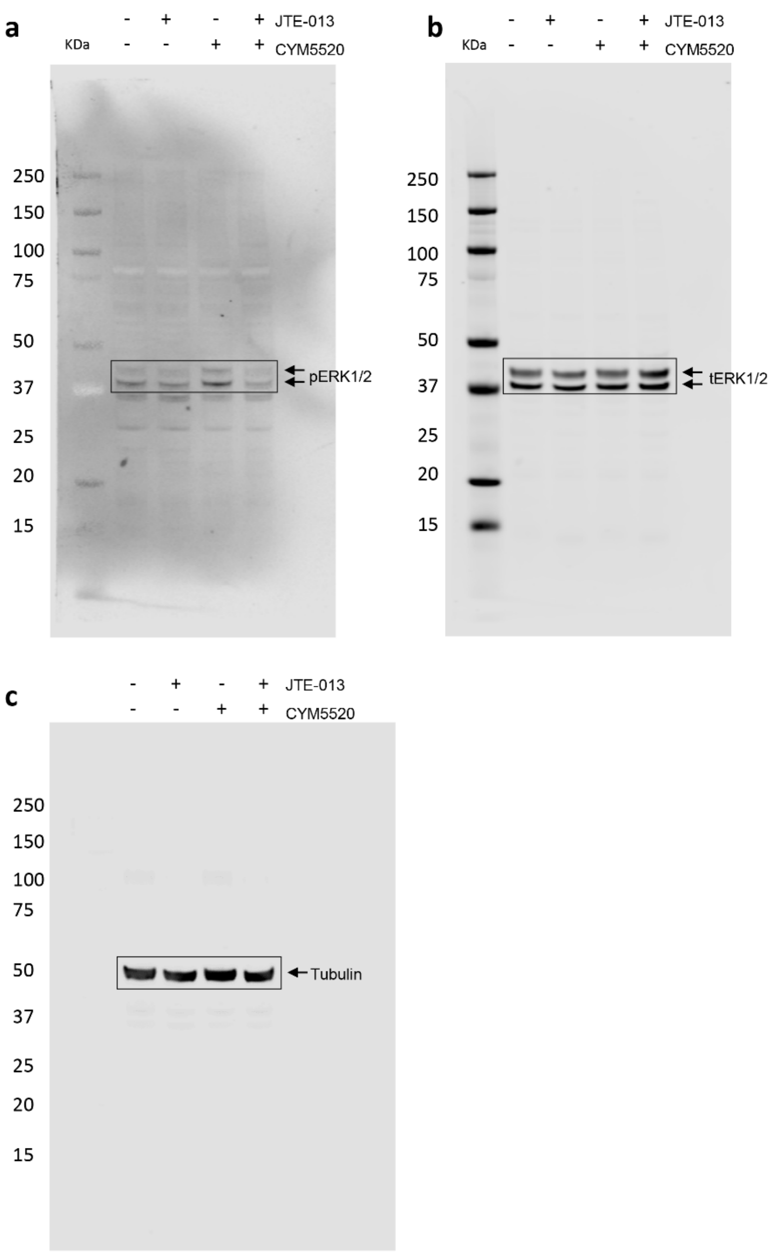

Supplemental Figure S2 – Full Blots of Figure 2D

To confirm agonism of $S1P_2$, HEK93T cell lysates from the treatment groups were subjected to SDS-PAGE and western blotting to detect a) mouse anti-phospho-ERK1/2, b) rabbit anti-total ERK with c) mouse anti-tubulin as a loading control on the same blots. Markers bands are detected in the same channel as the anti-rabbit antibody (700 nm) in b). Results are representative of 3 independent experiments.

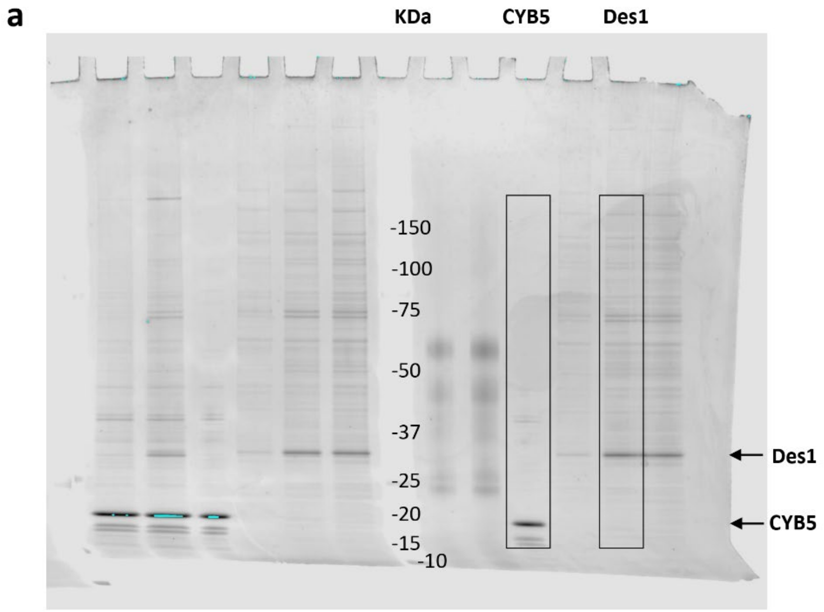

Supplemental Figure S3 – Full Gel of Figure 3C

a) Des1-FLAG was overexpressed in HEK293T cells for 24 h, purified using FLAG-agarose beads and eluted with FLAG peptide. Recombinant CYB5 (Sigma) and purified Des1-FLAG were separated by SDS-PAGE and detected by SYPRO-RUBY staining.

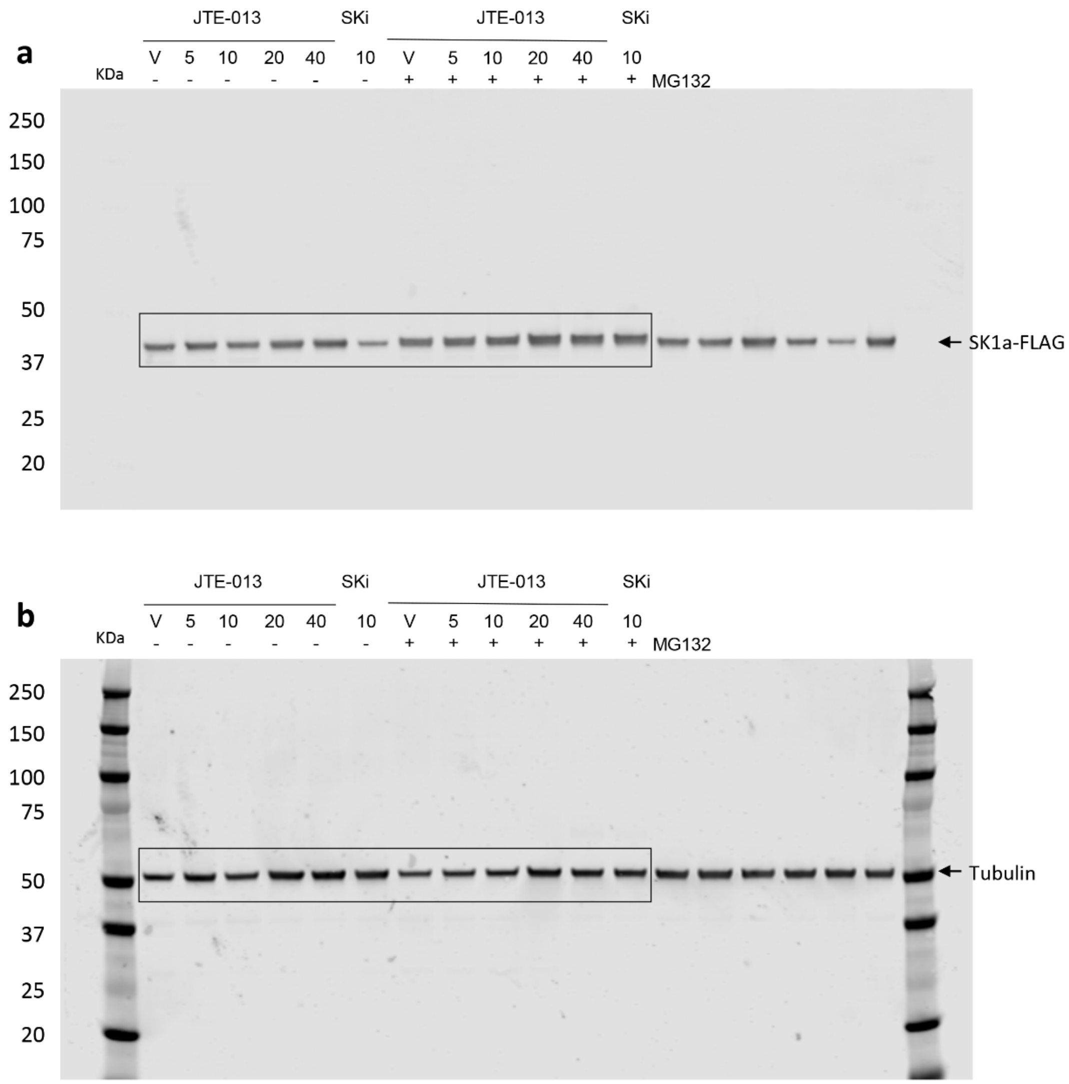

Supplemental Figure S4- Full Blots of Figure 4B

Western blots show the effect of varying concentrations of JTE-013 (5-40 μM) or 20 μM SKi for 24 h compared to vehicle (V) treatment in the presence or absence of 10 μM MG132 on SK1a-FLAG in HEK293 cells. a) Rabbit anti-FLAG blot for SK1-FLAG is shown b) Mouse anti-Tubulin was used as a loading control. Markers bands are detected in the same channel as the anti-mouse antibody (700 nm), sizes are shown in b). Results are representative of 3 independent experiments.
